# Airborne quantification of net methane and carbon dioxide fluxes from European Arctic wetlands in Summer 2019

**DOI:** 10.1098/rsta.2021.0192

**Published:** 2022-01-24

**Authors:** Patrick A. Barker, Grant Allen, Joseph R. Pitt, Stéphane J.-B. Bauguitte, Dominika Pasternak, Samuel Cliff, James L. France, Rebecca E. Fisher, James D. Lee, Keith N. Bower, Euan G. Nisbet

**Affiliations:** ^1^ School of Earth and Environmental Sciences, University of Manchester, Manchester M13 9PL, UK; ^2^ School of Marine and Atmospheric Sciences, Stony Brook University, 145 Endeavour Hall, Stony Brook, NY 11794-5000, USA; ^3^ FAAM Airborne Laboratory, National Centre for Atmospheric Sciences, Building 146, College Road, Cranfield MK43 0AL, UK; ^4^ Wolfson Atmospheric Chemistry Laboratories, Department of Chemistry, University of York, Heslington, York YO10 5DD, UK; ^5^ Department of Earth Sciences, Royal Holloway, University of London, Egham, Surrey TW20 0EX, UK; ^6^ British Antarctic Survey, Natural Environment Research Council, Cambridge CB3 0ET, UK

**Keywords:** methane, carbon dioxide, wetland, Arctic, aircraft, flux

## Abstract

Arctic wetlands and surrounding ecosystems are both a significant source of methane (CH_4_) and a sink of carbon dioxide (CO_2_) during summer months. However, precise quantification of this regional CH_4_ source and CO_2_ sink remains poorly characterized. A research flight using the UK Facility for Airborne Atmospheric Measurement was conducted in July 2019 over an area (approx. 78 000 km^2^) of mixed peatland and forest in northern Sweden and Finland. Area-averaged fluxes of CH_4_ and carbon dioxide were calculated using an aircraft mass balance approach. Net CH_4_ fluxes normalized to wetland area ranged between 5.93 ± 1.87 mg m^−2^ h^−1^ and 4.44 ± 0.64 mg m^−2^ h^−1^ (largest to smallest) over the region with a meridional gradient across three discrete areas enclosed by the flight survey. From largest to smallest, net CO_2_ sinks ranged between −513 ± 74 mg m^−2^ h^−1^ and −284 ± 89 mg m^−2^ h^−1^ and result from net uptake of CO_2_ by vegetation and soils in the biosphere. A clear gradient of decreasing bulk and area-averaged CH_4_ flux was identified from north to south across the study region, correlated with decreasing peat bog land area from north to south identified from CORINE land cover classifications. While N_2_O mole fraction was measured, no discernible gradient was measured over the flight track, but a minimum flux threshold using this mass balance method was calculated. Bulk (total area) CH_4_ fluxes determined via mass balance were compared with area-weighted upscaled chamber fluxes from the same study area and were found to agree well within measurement uncertainty. The mass balance CH_4_ fluxes were found to be significantly higher than the CH_4_ fluxes reported by many land-surface process models compiled as part of the Global Carbon Project. There was high variability in both flux distribution and magnitude between the individual models. This further supports previous studies that suggest that land-surface models are currently ill-equipped to accurately capture carbon fluxes inthe region.

This article is part of a discussion meeting issue 'Rising methane: is warming feeding warming? (part 2)'.

## 1. Introduction

As of 2020, atmospheric abundances of the greenhouse gases methane (CH_4_) and carbon dioxide (CO_2_) have increased by approximately 1155 ppb and 132 ppm, respectively, since 1850 AD, and continue to rise at an estimated rate of 9 ppb per year for CH_4_ and 2 ppm per year for CO_2_ [1]. The global atmospheric emission budgets of both CH_4_ and CO_2_ still remain uncertain, with the balance between total anthropogenic and biospheric sources and sinks yet to be fully understood and accounted for. A temporary stagnation in CH_4_ growth between 1998 and 2007 [2], and renewed growth with a concurrent shift in carbon-13 isotopic ratio to lighter bulk abundance since 2007, further compound the current uncertainties associated with CH_4_ source and sink apportionment [3–5].

Wetlands are understood to be a key ecosystem in terms of the surface exchange of climate-relevant trace gases. CH_4_ is produced by methanogenic archaea under anoxic conditions with high soil organic carbon (SOC) in wetland soils. The magnitude of CH_4_ production within wetland soils is highly sensitive to temperature, SOC availability, presence of vegetation, and water table height and hence oxygen content of the soil [6–9]. Consequently, the number of variables affecting CH_4_ production, as well as their spatial and temporal variability, cause significant difficulty in parametrizing and predicting current and future CH_4_ emissions from wetlands accurately [10]. Global wetlands are thought to represent the largest single natural source of atmospheric CH_4_, contributing approximately 101–179 Tg CH_4_ yr^−1^ to the global CH_4_ budget which represents 20% of the global yearly CH_4_ source to the atmosphere [9,11,12]. In addition to producing CH_4_, well-drained mineral soils under aerobic conditions can facilitate oxidation of CH_4_ to CO_2_ by methanotrophic microorganisms [13], while the surface exchange of CO_2_ is controlled by the balance between respiratory CO_2_ production from soil carbon stocks and photosynthetic CO_2_ uptake by vegetation [14]. The Arctic is currently a net CO_2_ sink, with an average of −0.13 Pg CO_2_ year^−1^ taken up by the terrestrial arctic; this CO_2_ sink is highest in the summer months, when gross primary productivity is at a maximum [15]. Recent research has identified that longer Arctic growing seasons, increased precipitation and evapotranspiraton rates may be driving increases in the Arctic CO_2_ sink magnitude. Conversely, higher ecosystem respiration rates and hence CO_2_ emission have been linked to higher air temperatures in the Arctic. It is therefore evident that the rapidly changing climate in the Arctic has the potential to significantly impact the source–sink dynamics of CO_2_ exchange in this area, and continuous *in situ* monitoring is crucial to assess the impact of climate change on Arctic CO_2_ fluxes [16–18]

Approximately 53% of global wetland area is situated in northern latitudes above 50°N [19]. Therefore, Arctic and Boreal wetlands contribute significantly to the global CH_4_ budget [20]. In addition to the current high CH_4_ emission from high-latitude wetlands, these areas are sensitive to increasing CH_4_ emission from positive climate feedbacks and Arctic climate amplification. Arctic mean air temperatures have increased at more than twice the rate of the global average, with current arctic temperature growth over 1.5°C higher than the 1971–2000 global average temperature growth with further warming predicted for the future [21,22]. Higher temperature may result in increased microbial activity in wetland ecosystems, leading to enhanced methanogenesis [23]. Furthermore, thawing of permafrost as a result of increasing temperature may result in an increase in arctic wetland extent as well as enabling the release of organic carbon from the estimated approximately 1700 Pg of stored SOC in arctic permafrost [24–27]. It is therefore clear that the CH_4_ emissions from high-latitude wetlands may become increasingly important over time due to their high sensitivity to climate change.

Wetland trace gas emissions are commonly determined via top-down inversion modelling, bottom-up process-based modelling [7], or upscaling of eddy covariance or chamber fluxes to a wider wetland area. [28,29]. Process modelling of CH_4_ fluxes from the wider Arctic often feature large uncertainty ranges due to the highly complex set of variables that influence microbial CH_4_ production and emission processes to atmosphere. Additionally, the spatial and temporal heterogeneity of wetland environments, as well as the poorly defined boundaries of wetlands that often change seasonally, add significant uncertainty to annualized emission estimates as process models often account poorly for fine spatial and temporal variability in emissions [7,9]. In situ measurements of wetland emissions can be used to evaluate and improve process model estimates. However, the majority of *in situ* flux measurements are on a much smaller spatial scale than typical model outputs (typically on a 0.5° grid), and there are currently few *in situ* measurements on an appropriate scale for more direct model comparison [29,30]. Aircraft measurement platforms allow *in situ* measurements of trace gas emissions to be carried out on a similar spatial scale to process models, albeit as discrete snapshots of flux, and may allow the uncertainties on emission estimates from such models to be better constrained [31–33].

The Methane Observations and Yearly Assessments (MOYA) project aimed to use *in situ* measurements, targeted field campaigns and modelling to constrain global CH_4_ sources and sinks from a variety of key CH_4_ emission hotspots, such as African biomass burning [34] and Tropical wetlands (Shaw *et al*. in review). In situ measurements of CH_4_ fluxes in these key areas will aid in reducing the uncertainty in their contribution to the global CH_4_ budget and may provide a clearer explanation for currently rising atmospheric CH_4_ mole fractions (MFs). As part of the MOYA project, the MOYA-Arctic field campaign was conducted from 29 July 2019 to 2 August 2019 based in Kiruna, Sweden. This field campaign used *in situ* aircraft measurements to quantify emissions of CH_4_ and other trace gases from northern Swedish and Finnish (Fennoscandian) wetlands (66–69°N, 22–28°E) during the summer period. This work presents *in situ* aircraft measurements of CH_4_, CO_2_ and N_2_O MF during one of the survey flights carried out during the MOYA-Arctic campaign. From these measurements, mass balance flux estimates for CH_4_ and CO_2_ were calculated and compared with previous similar aircraft studies in the region by O'Shea *et al*. [33]. Despite no direct flux being attainable from the N_2_O data, a minimum flux threshold using this mass balance method was calculated for N_2_O. Additionally, this study compares the fluxes obtained via aircraft mass balance with fluxes from Global Carbon Project (GCP) wetland process models, where both the magnitude and spatial distribution of CH_4_ fluxes are compared with the aircraft results.

## 2. Methods

### (a) Airborne instrumentation

The FAAM BAe 146-301 Atmospheric Research Aircraft (FAAM ARA) was operated for *in situ* sampling during the MOYA-Arctic campaign. Thermodynamic and meteorological parameters such as temperature, pressure and three-dimensional wind vector were measured by the FAAM ARA core instrument suite [35]. Temperature was measured by a Rosemount 102 sensor, with an estimated uncertainty of 0.1 K. Static pressure was measured by a series of pitot tubes distributed across the aircraft surface, with an uncertainty of 0.3 hPa. The three-dimensional wind vector is measured by a nose-mounted five port turbulence probe, with an uncertainty of 0.2 m s^−1^.

A Los Gatos Research Fast Greenhouse Gas Analyser (FGGA) was used for 10 Hz measurements of CO2 and CH_4_ MF. The FGGA instrument uses a Cavity-Enhanced Absorption Spectroscopy technique and two continuous-wave near-IR diode lasers. The FGGA is mounted within a 19-inch rack in the cabin of the aircraft with ambient air pumped via a rearward-facing 3/8’ stainless steel inlet mounted to a window blank. The FGGA was calibrated using three calibration gas standards: high- and low-concentration calibrations to account for instrument drift over the course of a flight, and a target calibration to assess long-term instrument precision and bias over multiple flights. All three calibration standards were traceable to the National Oceanic and Atmospheric Administration/Earth System Research Laboratory (NOAA/ESRL) World Meteorological Organisation (WMO) X2004A scale for CH_4_ and X2007 scale for CO_2_ [34,36]. Accounting for all sources of uncertainty, the mean (calibrated) biases and associated 1*σ* overall uncertainties are estimated to be −0.048 ± 0.626 ppm and −1.22 ± 2.93 ppb, respectively for 10 Hz CO_2_ and CH_4_ sampling during MOYA-Arctic. One hertz measurements of N_2_O MF were sampled by an Aerodyne Quantum Cascade Laser Absorption Spectrometer (QCLAS). The QCLAS was calibrated by means of three calibration gas standards, which were traceable to the WMO X2006 calibration scale [34,37]. An overall 1*σ* uncertainty of 0.58 ppb was estimated for 1 Hz N_2_O MF measurements during the MOYA-Arctic flights.

### (b) Aircraft mass balance flux technique

Aircraft mass balance flux techniques are well established in their ability to quantify trace gases fluxes from various sources, including regional-scale city emissions [38–40], point-source oil and gas emissions [41–43] and regional-scale biospheric trace gas emission/uptake [33,44]. For reliable flux quantification using aircraft mass balance, several criteria must be satisfied. First, MF measurements must be made downwind of a targeted emission source. Second, background measurements should be made, either within the centre of the well-mixed boundary layer upwind of the targeted emission source, or from downwind measurements either side of the emissions plume from the targeted emission source. These background measurements represent an estimate of the MF that would have been measured downwind of the targeted source in the absence of any emissions from that source. Additionally, wind direction should ideally be perpendicular to upwind and downwind sampling to ensure the measured airmass advects over the emission source, and wind speed should be constant for mass balance calculations. The meteorological conditions at the time of the survey flight reported here were highly favourable for this approach and the survey design was optimized to sample accordingly (described in §3). Flux determination by aircraft mass balance is expressed by equation (2.1).

2.1 $$\mathrm{Flux}=\int_{0}^{z}\int_{x_{0}}^{x_{i}}(C_{\mathrm{Enh}}-C_{0})U_{⊥}dxdz$$

and

2.2 $$C_{\left({\mathrm{gm}}^{-3}\right)}=\frac{MF_{\left(\mathrm{ppb}\right)}}{10^{9}}\times \rho_{\mathrm{air}}\times \frac{M_{x}}{M_{\mathrm{air}}}\;\;x={\mathrm{CH}}_{4}\;\;\text{or}\;\;{\mathrm{CO}}_{2}$$

The flux of a trace gas species in g s^−1^ is defined as the enhancement in trace gas concentration (*C*_Enh_ is the enhanced concentration downwind in this case, *C*_0_ is the background concentration). MFs are first converted to concentrations in units of g m^−3^ using equation (2.2), where *ρ*_air_ is the molar density of air, *M*_x_ is the molar mass of CH_4_ or CO_2_ and *M*_air_ is the molar mass of air. (*C*_Enh_ – *C*_0_) is then multiplied by the windspeed perpendicular to the flight track in m s^−1^, *U*_⊥_, integrated over the length of the downwind flight transect, *x*, and the height of the convective boundary layer, *z*. Measured statistical variability in the background concentration and wind vector, as well as measurement uncertainty and quantified systematic uncertainty in the height of boundary layer mixing (diagnosed from thermodynamic profiles), are propagated through equation (2.1) to determine flux uncertainty [33]. In addition to mass balance flux techniques, the FAAM ARA is capable of quantifying trace gas fluxes using the eddy covariance technique [45,46]; however, the magnitude of vertical windspeed during flight C195 was not sufficient for reliable calculation of CH_4_ or CO_2_ fluxes using eddy covariance in this study.

### (c) Chamber fluxes

The mass balance fluxes derived from airborne measurements have also been compared to area-weighted chamber flux measurements, which were carried out in the same study area investigated here as part of the CH_4_ and other greenhouse gases in the Arctic—Measurements, process studies and Modelling (MAMM) project. These chamber experiments were carried out daily between 12 July 2012 and 2 August 2012 and yielded area fluxes by specific land type for wetland (4.5 ± 3.7 mg m^−2^ h^−1^) and forest (0.05 ± 0.07 mg m^−2^ h^−1^) for summer. These area fluxes were scaled using the total wetland and forested area fraction with each of the three flux areas surveyed here according to the CORINE land cover map. The total wetland area was calculated as the sum of the peat bog and inland marsh grid cells within each area, and the total forested area was determined as the sum of all forest subclasses (broadleafed, coniferous and mixed forest) cells for each area. The chamber area fluxes were then multiplied by the total wetland or forest areas to give a bulk flux value for each of the three distinct flux areas.

### (d) Flight description and strategy

**Figure 1.**
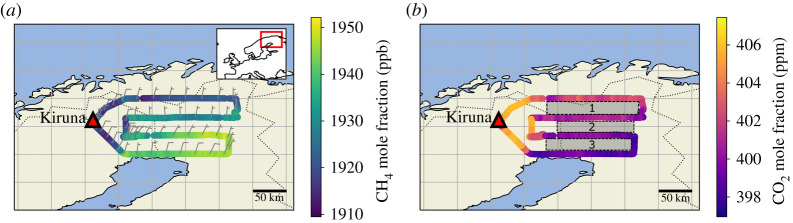
Flight track of FAAM flight C195 over northern Fennoscandian wetland areas; the flight tracks are coloured by (*a*) CH_4_ MF and (*b*) CO_2_ MF. Wind barbs are shown at 5 min intervals on (*a*) and Areas 1, 2 and 3 are shown on (*b*). (Online version in colour.)

The target area of FAAM ARA Flight C195 (figure 1) is mostly Northern Finnish Lapland, but also encompasses parts of Northeast Sweden (Norbotten County) and North Norway (Finnmark County). The area surveyed was comprised boreal (Taiga) forest interspersed with peat bogs and lakes. Seasonal thaw of accumulated winter snow and ice typically results in the high prevalence of semi-permanent water bodies and peatland mires in the summer months. Flight C195 was carried out on 31 July 2019 between 10 : 00 and 14 : 30 CEST and involved four straight aircraft transects of approximately 200 km length across the wetland area, each at constant latitude. The first of these transects was the northernmost upwind leg at 69°N latitude, and the legs step down southwards in increments of 1°N with the final southernmost downwind leg at 66°N (as shown in figure 1). These constant latitude transects at 69°N, 68°N, 67°N and 66°N are referred to as transects 1, 2, 3 and 4 throughout. All transects across the wetland were conducted at altitudes between 300 m and 600 m above ground level (agl). Six deeper profiles (three ascents, three descents) from approximately 300 m agl to approximately 2500 m agl were carried out at the start, middle and end of the flight in order to assess planetary boundary layer (PBL) height and development used to derive mixing height for equation (2.1) over the course of the sampling period. A single biomass burning plume was intercepted at approximately 12:22 CEST, but this was removed from the CO_2_ and CH_4_ data prior to flux calculations. Measurements of carbon monoxide (CO) remained constant during the flight (with the exception of the single fire plume), strongly suggesting that this biomass burning event as well as any other anthropogenic sources did not have any impact on CH_4_ MFs further downwind.

## 3. Results and discussion

### (a) Methane and carbon dioxide fluxes

Figure 1 shows the CH_4_ and CO_2_ MF variability over the course of flight C195. It can be seen that CH_4_ MF increases towards the southernmost extent of the flight track, with an approximate 40 ppb increase in CH_4_ between transect 1 and transect 3, with smaller MF increases between the southernmost transect 3 and transect 4. The isotopic signature of the CH_4_ emissions during this flight strongly suggests that the CH_4_ originates from a wetland source (see electronic supplementary material). The CO_2_ MF decreases by approximately 5 ppm between transect 1 and transect 4, consistent with net biospheric CO_2_ uptake over the survey area. There was no significant gradient in N_2_O MF observed over the course of flight C195 so N_2_O mass balance fluxes could not be calculated (see electronic supplementary material, figure S2). However, a theoretical ‘limit of detection’ for N_2_O mass balance fluxes using the aircraft instrumentation was derived using the standard deviation of the N_2_O MF over transect 1, and this is detailed in the electronic supplementary material.

**Figure 2.**
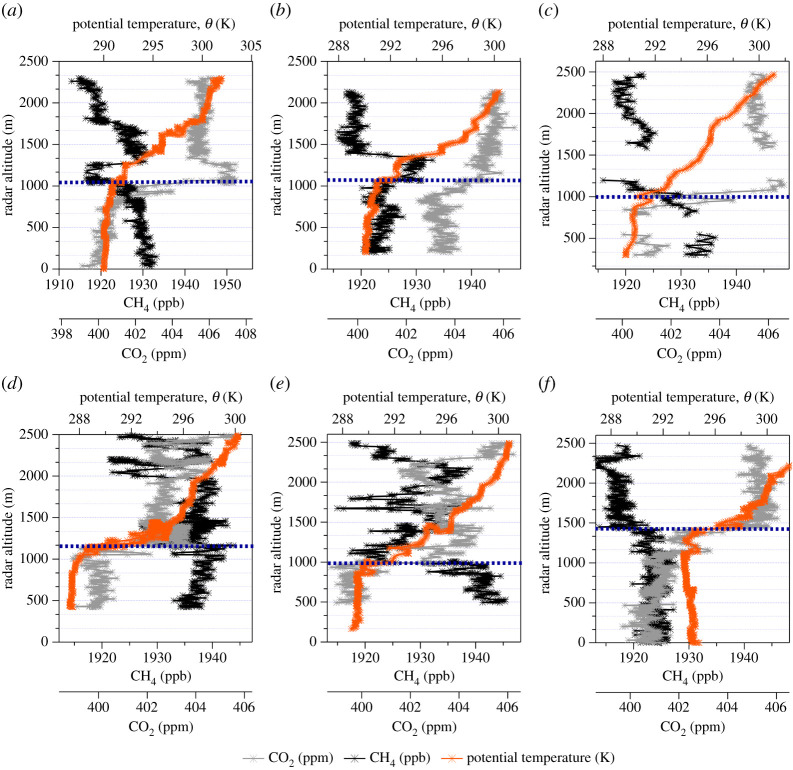
Vertical profiles of potential temperature, CH_4_ mixing ratio and CO_2_ mixing ratio during the six profiles (labelled chronologically (*a*–*f*)) carried out by the FAAM ARA during flight C195. Approximate convective mixing heights, determined by a change in vertical gradient in potential temperature, for each profile are also displayed as blue dashed lines. (Online version in colour.)

Figure 2 shows the potential temperature (*θ*), CH_4_ and CO_2_ MFs during the six altitude profiles carried out in flight C195. All profiles were conducted within the near vicinity of the study area at the start, middle and end of the flight, and the profiles bracket the four longitudinal transects across the study area (see electronic supplementary material, figure S3). There was very little change in PBL height between the first and second set of vertical profiles as diagnosed from the characteristic sharp change in potential temperature gradient seen at PBL top (dashed blue lines in figure 2). However, there is a significant difference between the final profile ascent (figure 2*e*) and the final profile descent (figure 2*f*), as the PBL height is observed to be approximately 1000 m agl, whereas the descent shows a PBL height approximately 450 m higher at approximately 1450 m AGL, this final descent profile is therefore not used in PBL determination for mass balance calculations. To account for this change in mixing height used in the mass balance approach, the nearest available thermodynamic profile to each transect was used to determine PBL height in the flux calculations (i.e. only the ascending profile in figure 2*e* is used). The relatively small increase in PBL height over the course of flight C195 suggests that any entrainment of free tropospheric air into the PBL can be considered to be negligible and therefore will not significantly affect the uncertainty of flux estimates calculated here. Furthermore, MFs of CH_4_ and CO_2_ within the PBL were observed to be constant within each of the deep profiles, suggesting that the PBL was well mixed throughout the study region.

**Figure 3.**
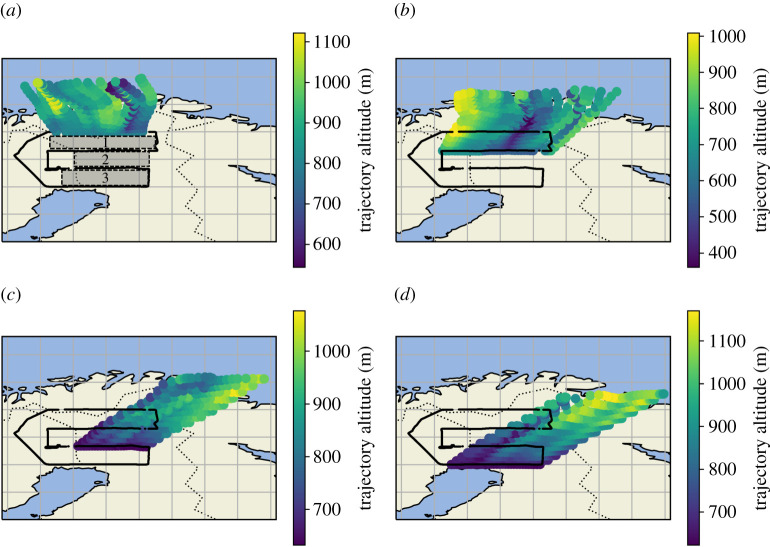
HYSPLIT 12 h back-trajectories coloured by trajectory altitude run every 60 s from each constant latitude leg. Areas 1, 2 and 3 are shown on (*a*). (Online version in colour.)

Wind direction over the course of flight C195 was predominantly northerly during transect 1 and transect 2 as shown in table 1 and by the wind barbs in figure 1*a*. As the flight progressed, the average wind direction changed from northerly to more north-easterly winds towards the southern end of the flight track was also confirmed by HYSPLIT back-trajectories with trajectory endpoints calculated for each transect shown in figure 3. Owing to this gradual change in wind direction over the course of the flight, a mass balance flux calculation across the entire flight area (i.e. using transect 1 as the background and transect 4 as the enhanced run) would be inappropriate, as transect 1 does not sample the same airmass as transect 4. Therefore individual fluxes were calculated between parallel meridional transect pairs, with the northern transect of each pair used to determine the upwind background, and the southern transect to determine the CH_4_ gradient over the distance between each pair. The three areas between the meridional transect pairs are referred to as Area 1, Area 2 and Area 3 throughout. Area 1 is between transect 1 (69°N) and transect 2 (68°N), Area 2 is between transect 2 (68°N) and transect 3 (67°N), and Area 3 is between transect 3 (67°N) and transect 4 (66°N).

**Table 1.** Aircraft mass balance CH_4_ and CO_2_ flux parameters for flight C195. Total fluxes and hourly area fluxes within three distinct flux areas enveloped by a southern background transect and northern enhanced transect are also included for CH_4_ and CO_2_. CH_4_ area fluxes are reported normalized to the total land area within each of the three study areas, and also normalized to the total wetland area within each study area.

parameter	Area 1 (transect 1 - transect 2)	Area 2 (transect 2 - transect 3)	Area 3 (transect 3 - transect 4)
CH_4_ enhancement over background (ppb)	9.98	11.12	1.65
CO_2_ enhancement over background (ppm)	−1.19	−1.08	−0.96
(C_Enh_ – C_0_) (CH_4_)	6.37 × 10^−6^ ± 2.98 × 10^−10^ g m^−3^	7.32 × 10^−6^ ± 3.88 × 10^−10^ g m^−3^	1.08 × 10^−6^ ± 3.87 × 10^−10^ g m^−3^
(C_Enh_ – C_0_) (CO_2_)	−2.11 × 10^−3^ ± 1.87 × 10^−7^ g m^−3^	−1.94 × 10^−3^ ± 1.66 × 10^−7^ g m^−3^	−1.72 × 10^−3^ ± 1.78 × 10^−7^ g m^−3^
perpendicular windspeed, U_⊥_	5.61 ± 1.32 m s^−1^	4.19 ± 1.32 m s^−1^	7.63 ± 1.09 m s^−1^
mean wind direction	183°	207°	196°
transect Length, x	2.28 × 10^5^ m	1.93 × 10^5^ m	2.17 × 10^5^ m
boundary layer height, z	962 m	1073 m	1202 m
CH_4_ scaled chamber flux (O'Shea *et al*.)	6.86 ± 5.75 kg s^−1^	5.04 ± 4.26 kg s^−1^	2.48 ± 2.20 kg s^−1^
CH_4_ flux (emission rate)	7.85 ± 1.06 kg s^−1^	6.37 ± 2.01 kg s^−1^	2.15 ± 0.31 kg s^−1^
CH_4_ hourly area flux (total land area)	1.11 ± 0.26 mg m^−2^ h^−1^	1.07 ± 0.34 mg m^−2^ h^−1^	0.32 ± 0.046 mg m^−2^ h^−1^
CH_4_ hourly area flux (wetland area)	5.31 ± 0.72 mg m^−2^ h^−1^	5.93 ± 1.87 mg m^−2^ h^−1^	4.44 ± 0.64 mg m^−2^ h^−1^
CO_2_ flux (emission rate)	−2601 ± 615 kg s^−1^	−1692 ± 533 kg s^−1^	−3431 ± 493 kg s^−1^
CO_2_ hourly area flux (total land area)	−369 ± 87 mg m^−2^ h^−1^	−284 ± 89 mg m^−2^ h^−1^	−513 ± 74 mg m^−2^ h^−1^

Table 1 shows the total CH_4_ and CO_2_ fluxes calculated for flight C195. Area-normalized fluxes are presented in units of mg m^2^ h^−1^ for comparison with fluxes reported by process models (see §3.3). The CH_4_ area fluxes calculated in this work agree well with previous analogous studies in the region in Arctic summer. For example, O'Shea *et al*. calculated CH_4_ and CO_2_ fluxes using aircraft mass balance in a similar study area of northern Sweden and Finland [33]. The CH_4_ flux of 1.2 ± 0.5 mg m^−2^ h^−1^ reported by O'Shea *et al*. agrees within overlapping 1*σ* uncertainty for the Area 1 and Area 2 fluxes derived during this work (1.11 ± 0.26 mg m^−2^ h^−1^ for Area 1 and 1.07 ± 0.34 mg m^−2^ h^−1^ for Area 2) but agrees poorly with the CH_4_ flux of 0.32 ± 0.26 mg m^−2^ h^−1^ for Area 3. The O'Shea *et al*. study involved a July 2012 aircraft survey in the same region as Areas 1 and 2, which explains why fluxes from these areas agree best with the O'Shea *et al*. results. From the fluxes presented in this work and previous fluxes reported for the same area, it appears that CH_4_ emission in this area of the Arctic has not increased significantly from the period 2012–2019. However, climatological data from within the study area in Sodankylä shows that both 2012 and 2019 had similar July average temperatures (13.6°C for 2012 and 13.3°C for 2019), which may account for some of the similarity between the CH_4_ fluxes. In addition, both July average temperature and precipitation for 2012 and 2019 are below the average for the period 1981–2010 (14.3°C, 73 mm), which suggests that CH_4_ fluxes could be higher in years where temperature and precipitation anomalies are higher [47].

The net CO_2_ uptake observed during this study is higher than that reported in previous work. The CO_2_ sink reported from Arctic wetlands by O'Shea *et al*. is −350 ± 143 mg m^−2^ h^−1^, which agrees within overlapping 1*σ* uncertainty for CO_2_ area fluxes reported here for each area (table 1), despite the maximum average CO_2_ flux value calculated in this study being 24% higher than that determined in O'Shea *et al*. However, the Christensen *et al*. chamber CO_2_ flux of −96 ± 33 mg m^−2^ h^−1^ is significantly lower than the CO_2_ area fluxes for Area 1 and Area 2 [24]. Biospheric CO_2_ fluxes are known to exhibit strong spatio-temporal variability that is highly sensitive to temperature, precipitation, insolation and leaf area index of the vegetation types studied, and therefore a close agreement between studies conducted on different days and years is not expected.

**Figure 4.**
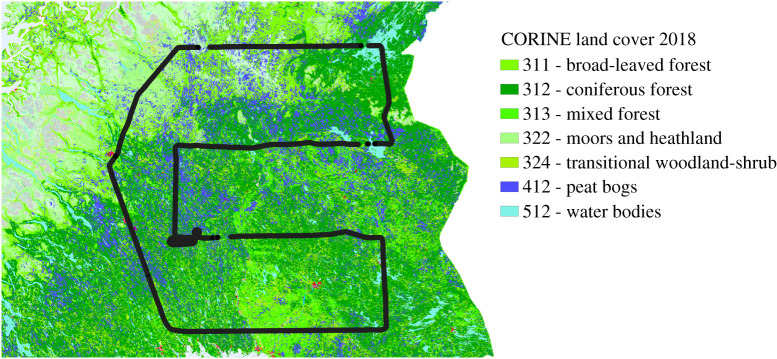
CORINE 2018 land cover map of the northern European wetland area surveyed during flight C195. The flight track is also displayed. (Online version in colour.)

Table 1 shows that the mean CH_4_ emission rate and area flux decreases with decreasing latitude from Area 1 to Area 3. Figure 4 shows the 2018 Copernicus Land Monitoring Service CORINE land cover classification of the study area (https://land.copernicus.eu/pan-european/corine-land-cover/clc2018), and table 2 shows the most abundant land classes within each flux area by percentage. It can be seen from both table 2 and visually from figure 4 that the abundance of peat bogs decreases towards the south of the survey area. Peat bogs comprise 22.9% of the land cover within the northernmost Area 1 between transect 1 and transect 2, this decreases slightly to 20.0% within Area 2 and decreases further to 7.28% within the southernmost Area 3. The decreasing peat bog abundance towards the southern end of the survey area provides a likely explanation for the gradually decreasing CH_4_ flux seen from north to south in table 2. Additionally, there is a positive correlation between CO­_2_ sink magnitude and CORINE vegetation cover within the three areas of the flight (electronic supplementary material, figure S5). However, the correlation between CO_2_ sink and vegetation cover is weaker than the CH_4_ flux-peatland area correlation. This is likely due to the differing CO_2_ uptake capacities of specific vegetated land types (e.g. dense forest will sequester more CO_2_ than an equivalent area of cropland).

**Table 2.** Top 5 CORINE land cover classes by percentage for each mass balance flux box.

Area 1		Area 2		Area 3	
land class	percentage cover	land class	percentage cover	land class	percentage cover
coniferous forest	41.2	coniferous forest	42.8	coniferous forest	49.8
peat bogs	22.9	mixed forest	21.5	mixed forest	18.6
broad leaf forest	13.2	peat bogs	20.0	woodland shrub	16.4
heathland and moors	5.51	woodland shrub	11.2	peat bogs	7.28
mixed forest	4.93	water bodies	2.80	water bodies	5.06

**Figure 5.**
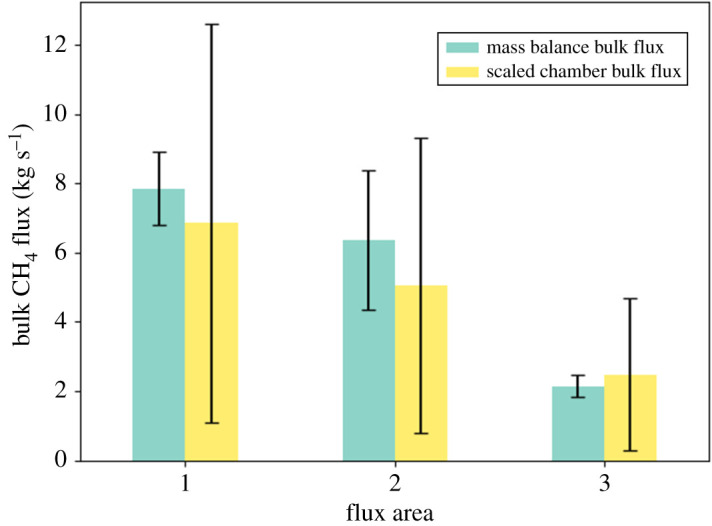
Bar plot comparison of bulk CH_4_ flux from the three flux areas for the mass balance method and the upscaled chamber fluxes from the MAMM project. Error bars indicate the 1-σ standard deviation flux uncertainty in each case. (Online version in colour.)

The mass balance fluxes of CH_4_ derived in this study are compared to upscaled chamber CH_4_ flux measurements that were previously taken in the same study area. A description of the chamber measurements as well as the method of upscaling these fluxes can be found in §2.3. The chamber flux results are shown in table 1 and figure 5. It can be seen that the scaled chamber fluxes have a larger relative error of between 84% and 88% of the flux value when compared to the mass balance fluxes (between 14% and 31%); however, the mean mass balance and chamber bulk fluxes agree very well within overlapping 1σ uncertainty for all three flux areas. The mean bulk fluxes from mass balance for Areas 1 and 2 (7.85 ± 1.06 kg s^−1^ and 6.37 ± 2.01 kg s^−1^, respectively) are approximately 15–25% higher than the scaled chamber fluxes of 6.86 ± 5.75 kg s^−1^ for Area 1 and 5.04 ± 4.26 kg s^−1^ for Area 2. The slightly higher fluxes from mass balance could be associated with the presence of plant-mediated wetland CH_4_ emission via the transport of CH_4_through specialized plant tissues. Emission from this pathway would be detectable using mass balance techniques but may be missed when using flux chamber apparatus mounted at ground level. However, the agreement between the two techniques provides support for the efficacy of the mass balance technique compared to ground-based flux quantification techniques and demonstrates the potential for spatial scalability and interpretation of point measurements such as chamber fluxes.

### (b) Comparison with land-surface model methane fluxes

Top-down *in situ* flux estimates such as those derived in this work can provide an important comparison to emission estimates from bottom-up biogeochemical process models and anthropogenic emission inventories and can provide crucial validation of such models. Wetland CH_4_ fluxes are typically derived by land-surface models by parametrizing key biogeochemical characteristics, such as CH_4_ production, transport and oxidation within wetland soils, as well as the amount and type of vegetation present. These initial parameters are then forced by environmental variables such as precipitation, temperature, respiration and atmospheric CO_2_ concentration in order to account for seasonal and interannual differences in CH_4_ emission [48,49]. The CH_4_ flux density output from biogeochemical parametrization is then combined with a wetland distribution map for a given area to spatially distribute the CH_4_ flux and produce a flux map [7]. Recent research by Saunois *et al*. has compiled monthly CH­_4_ flux data from 13 different land-surface wetland models over the period 2000–2017 as part of the GCP. These model outputs, along with top-down atmospheric inversions, have provided an updated estimate for the global CH_4_ budget for the 2000–2017 period. Mean modelled CH_4_ flux for every July month was taken from the years 2000–2017 to best represent the northern hemisphere summertime period corresponding to the flight C195 survey data. There was found to be no significant trend of increasing or decreasing CH_4_ flux reported by the models over the 2000–2017 period as shown in electronic supplementary material, figure S6. All land-surface models shown here use a diagnostic means of prescribing wetland cover, namely the Wetland Area Dynamics for Methane Modelling (WAD2 M) which uses satellite microwave remote-sensing inputs [50]. Seven of the 13 process models also include prognostic with internal wetland prescription in each model.

**Figure 6.**
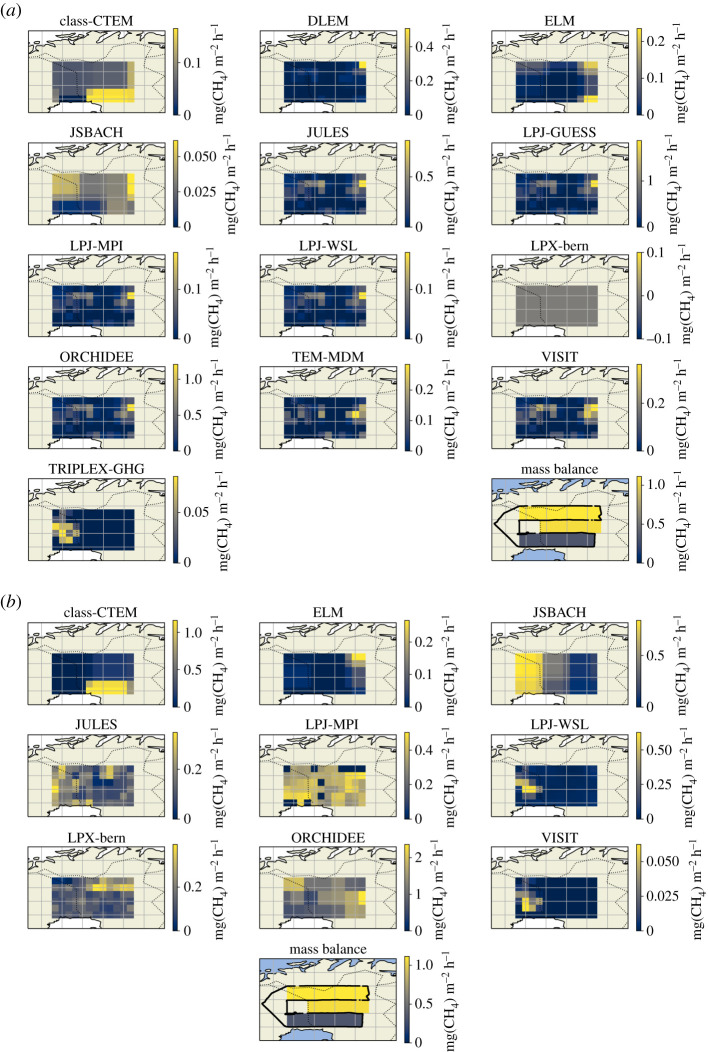
Maps of modelled CH_4_ flux in mg m^−2^ h^−1^ for the study area of flight C195 from various land-surface process models; (*a*) shows model outputs that have used the diagnostic WAD2M remote-sensing product to prescribe wetland cover and dynamics, (*b*) shows models that have used prognostic wetland cover information determined by the models themselves. The model data are obtained from the supplementary data of Saunois *et al*. [9]. Maps of the aircraft mass balance flux results are also shown in figure 6*a*,*b*. (Online version in colour.)

Figure 6*a,b* show modelled CH_4_ flux distribution maps for the diagnostic and prognostic models, respectively. The majority of diagnostic models share a common spatial distribution of CH_4_ flux due to the WAD2M product that these models use to prescribe wetland cover. This common flux pattern shows flux hotspots in the northern two-thirds of the study area, particularly to the northeast. The prognostic model outputs do not show a common pattern of CH_4_ flux distribution as with the diagnostic models, and flux distributions are much more variable in these model variants. The majority of diagnostic GCP models and a select few of the prognostic models (namely LPX-Bern and ORCHIDEE) show peak fluxes in the northern two-thirds of the study area, which broadly agrees with the mass balance flux observations where the highest fluxes were also measured in the northern two-thirds. Additionally, the aforementioned models also appear to show flux hotspots towards the eastern end of the study area. The mass balance technique could not explicitly resolve west to east flux gradient in this case; however, figure 1 appears to show higher CH_4_ MFs towards the eastern end of the flight track suggesting that CH_4_ fluxes may be higher towards this eastern end. Despite the differences and similarities in flux distribution between models and mass balance, it should be noted that the model outputs presented here are July averages over period of 17 years. It is therefore highly probable that wetland distribution in this area has changed over this time period, and good agreement between model flux distribution and mass balance flux distribution is not necessarily expected due to this.

**Figure 7.**
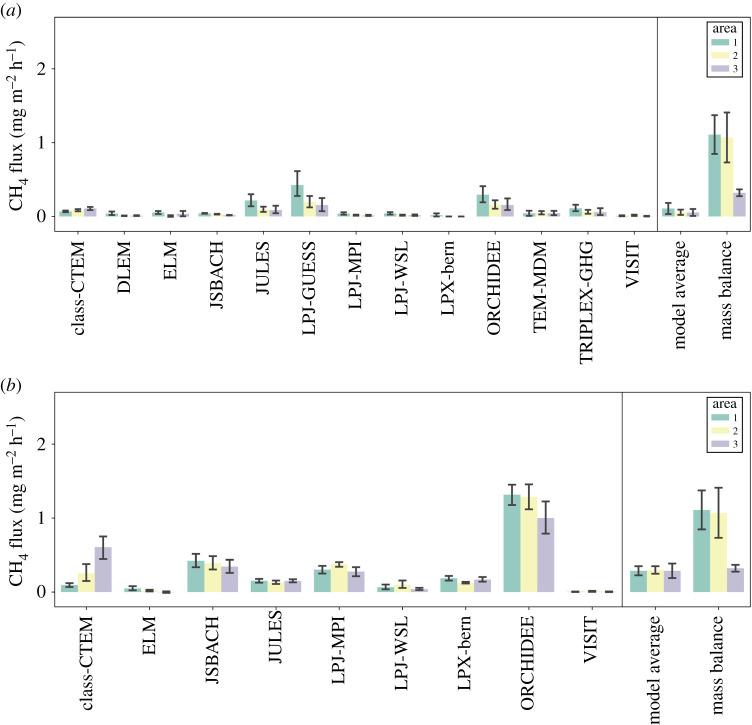
Bar plots of CH_4_ flux (in units of mg m^−2^ h^−1^) coloured by the three distinct flux areas of flight C195 from various land-surface process models and the aircraft mass balance results. Error bars indicate 1-σ standard deviations for the fluxes; (*a*) shows models with the diagnostic WAD2M wetland prescription and (*b*) shows models with the prognostic wetland cover information determined within the models. The model data are obtained from the supplementary data of Saunois *et al*. [9]. (Online version in colour.)

Figure 7 shows bar plots of the CH_4_ flux from the diagnostic and prognostic process models along with the mean mass balance fluxes from the three distinct flux areas identified in table 1. Most of the diagnostic and prognostic models report significantly lower CH_4_ fluxes for all three study areas compared to the mass balance results. In general, the prognostic models report higher CH_4_ fluxes for all three areas than the diagnostic models, most notably with the ORCHIDEE diagnostic model where fluxes for Areas 1 and 2 (1.32 ± 0.47 mg m^−2^ h^−1^ and 1.29 ± 0.54 mg m^−2^ h^−1^, respectively) agree well within overlapping uncertainty with the mass balance fluxes for Areas 1 and 2. Despite the general disagreement between modelled and mass balance CH_4_ fluxes in this case, it is worth noting that the mass balance results represent a single temporal snapshot from a single daytime flux from July 2019, whereas the model outputs are July monthly averages from 2000 to 2017. A likely source of disagreement between mass balance and process modelling in this case is that the mass balance may not be truly representative of the monthly average model output over multiple years. Having said this, an average air temperature of 12.2°C was measured on 31 July 2019 from the Sodankylä Lokka weather station during the time of the flight, which was slightly lower than the July mean temperature between 2000 and 2017 for the same weather station (14.5 ± 1.6°C). In addition, the average precipitation for July 2019 (33.4 mm) was also significantly lower than the July average precipitation between 2000 and 2017 (77.4 ± 28.2 mm) [51]. Lower temperature and precipitation for July 2019 suggest that the mass balance CH_4_ fluxes reported in this work may actually be lower than previous years, yet many of the GCP process models report significantly lower fluxes for the years previous to this study.

In summary, the land-surface models assessed here generally provide a lower estimate of wetland CH_4_ flux than top-down aircraft mass balance techniques for the study area of northern Sweden and Finland. However, the mass balance flux was measured during the daytime whereas the July average model outputs are comprised 24 h flux outputs. Wetland fluxes in the Arctic are known to exhibit a diurnal cycle with daytime flux maxima and night-time minima [52]; the inclusion of nocturnal low flux periods within the model outputs may partially account for the lower estimates of CH_4_ flux compared to mass balance. In addition to this, the GCP models only account for CH_4_ emission from areas classified as wetlands and do not account for lake, riverine or other biogenic CH_4_ sources. Mass balance will capture the flux footprint from all sources in the study area, not solely wetland. Therefore this could also account for the higher mass balance flux estimate relative to the model outputs, although isotopic analysis (electronic supplementary material, figure S4) suggests arctic peatlands are the primary CH_4_ source. Despite the previous points, there is still significant disagreement between individual model estimates of CH_4_ flux magnitude and distribution, and the models likely estimate lower CH_4_ flux even when taking the previous points into account. In order to provide model fluxes that are both more precise and more accurate, improvements in model inputs that more successfully estimate CH_4_ flux in comparison to *in situ* measurements, as well as standardized, accurate estimates of wetland cover and dynamics, are clearly both required. More frequent observational flux measurements are also ideally needed to provide important intercomparison and evaluation for model techniques. Put simply, the GCP models disagree markedly with one another, and with the measurements reported here for the region studied. It is imperative that this is addressed as a priority in order to more meaningfully use GCP models for Arctic carbon emissions, especially given the Arctic's rapidly changing climate.

## 4. Summary and Conclusion

A single research flight was carried out by the UK FAAM ARA across a wide area of northern European mixed peatland and forest. A peak wetland area-normalized flux of 5.93 ± 0.72 mg m^−2^ h^−1^ was obtained for CH_4_, and a peak total land area-normalized flux of −513 ± 74 mg m^−2^ h^−1^ was obtained for CO_2_ using the aircraft mass balance flux method for this area of northern Sweden and Finland (approximately 78 000 km^2^). The bulk CH_4_ fluxes determined via mass balance were found to agree well with upscaled chamber fluxes for the same study area. These results indicate that the wetlands in this area are a significant net source of CH_4_, and the area also represents a notable biospheric CO_2_ sink. A clear gradient of decreasing CH_4_ flux was identified between the northern and southern end of the flight track, which appears to correlate with decreasing peat bog land cover percentage from north to south. The mass balance fluxes were also compared with a variety of GCP land-surface process model fluxes, the majority of which were found to significantly underestimate CH_4_ emission in this area when compared to the mass balance. The results from this study provide an important wetland trace gas emission dataset that will aid validation of global land-surface models and will help further constrain the contribution of Arctic wetland and vegetation to global CH_4_ and CO_2_ budgets. Furthermore, the results highlight the sensitivity of bottom-up process models to accurate wetland cover and dynamics estimations and other input parameters when quantifying flux using these methods. This study also highlights an urgent need to improve land-surface models by using high-accuracy observational wetland cover datasets as model inputs, and by continuing *in situ* measurements as a means to evaluate the performance of these models. Continued improvements to land-surface models will allow them to more accurately predict summer CH_4_ emissions in the Arctic.

## Data accessibility

**Aircraft data:** FAAM ARA data from the MOYA project can be found on the CEDA archive (http://archive.ceda.ac.uk/) at https://catalogue.ceda.ac.uk/uuid/d309a5ab60b04b6c82eca6d006350ae6 (FAAM, NERC, Met Office. 2017).

**Basemap data:** The basemap used in figure 4 is obtained from ArcGIS software (Sources: Esri, HERE, Garmin, Intermap, Increment P Corp., GEBCO, USGS, FAO, NPS, NRCAN, GeoBase, IGN, Kadaster NL, Ordnance Survey, Esri Japan, METI, Esri China (Hong Kong). © OpenStreetMap contributors and the GIS User Community). The maps used in figures 1, 3, 6 and electronic supplementary material, figure S1 are produced using the Python Cartopy package, where maps are obtained from Natural Earth. Free vector and raster map data @ naturalearthdata.com.

**Land cover data:** The land cover maps used are obtained from the Copernicus Land Monitoring Service 2018 CORINE land cover dataset © European Union, Copernicus Land Monitoring Service 2021, European Environment Agency (EEA), f.ex. in 2018: © European Union, Copernicus Land Monitoring Service 2018, European Environment Agency (EEA).

**Model data:** The GCP model data used in this work and first reported by Saunois *et al*. [9] are obtained from the Integrated Carbon Observation System (ICOS) website (https://www.icos-cp.eu/GCP-CH4/2019, doi:10.18160/gcp-ch4-2019).

**Meteorological data:** Met data from the Sodankylä Lokka weather station were obtained from the Finnish Meteorological Institute (FMI) website (https://en.ilmatieteenlaitos.fi/download-observations)

The data are provided in the electronic supplementary material [53].
